# Amino acid deprivation induces AKT activation by inducing GCN2/ATF4/REDD1 axis

**DOI:** 10.1038/s41419-021-04417-w

**Published:** 2021-12-03

**Authors:** Hyeon-Ok Jin, Sung-Eun Hong, Ji-Young Kim, Se-Kyeong Jang, In-Chul Park

**Affiliations:** 1 KIRAMS Radiation Biobank, Korea Institute of Radiological and Medical Sciences, Seoul, 01812 Republic of Korea; 2 Division of Fusion Radiology Research, Korea Institute of Radiological and Medical Sciences, Seoul, 01812 Republic of Korea

**Keywords:** Cancer metabolism, Cell death

## Abstract

Amino acid availability is sensed by various signaling molecules, including general control nonderepressible 2 (GCN2) and mechanistic target of rapamycin complex 1 (mTORC1). However, it is unclear how these sensors are associated with cancer cell survival under low amino acid availability. In the present study, we investigated AKT activation in non-small cell lung cancer (NSCLC) cells deprived of each one of 20 amino acids. Among the 20 amino acids, deprivation of glutamine, arginine, methionine, and lysine induced AKT activation. AKT activation was induced by GCN2/ATF4/REDD1 axis-mediated mTORC2 activation under amino acid deprivation. In CRISPR-Cas9-mediated REDD1-knockout cells, AKT activation was not induced by amino acid deprivation, indicating that REDD1 plays a major role in AKT activation under amino acid deprivation. Knockout of REDD1 sensitized cells cultured under glutamine deprivation conditions to radiotherapy. Taken together, GCN2/ATF4/REDD1 axis induced by amino acid deprivation promotes cell survival signal, which might be a potential target for cancer therapy.

## Introduction

Amino acids are required substrates for protein synthesis and thus for cell proliferation [1]. Mammalian cells cannot synthesize all amino acids required for protein synthesis and therefore strictly depend on the availability of amino acids in their extracellular environment to support growth [1]. Central to the ability of a cell to adapt to its microenvironment is mechanistic target of rapamycin complex 1 (mTORC1), a critical signaling hub that is conserved from yeast to humans, regulating both growth and metabolism [2]. In the presence of amino acids, mTORC1 is translocated to the cytoplasmic surface of lysosomes, where it can be activated by growth factor signaling [3]. Upon activation, it stimulates amino acid uptake and protein synthesis by phosphorylating multiple targets [4]. Amino acid deprivation renders mTORC1 inactive and results in a reduction in protein synthesis rates [1]. Meanwhile, cells exhibit increased transport of extracellular and intracellular proteins to lysosomes, where their proteolytic degradation provides cells with an alternative supply of free amino acids [5].

During conditions of amino acid deprivation, the accumulation of uncharged tRNAs activates general control nonderepressible 2 (GCN2) [6]. When activated, GCN2 phosphorylates the eukaryotic initiation factor 2α subunit of the translation initiation complex, which in turn suppresses global translation while promoting the translation of select mRNAs, including the mRNA of the stress-responsive transcription factor, activating transcription factor 4 (ATF4) [7, 8]. ATF4 is the master transcriptional regulator of amino acid metabolism and coordinates the expression of critical metabolic genes, which increases nonessential amino acid synthesis as well as amino acid uptake, promoting cell survival and proliferation in amino acid-limited conditions [9, 10]. Loss of ATF4 suppresses cancer progression, suggesting its critical role in their survival by maintaining amino acid pools in cancer cells [10]. Indeed, many human cancers show activation of the GCN2/ATF4 pathway and depend on it to grow in nutrient-limited environments [10]. As a common mediator of various stress responses, including amino acid restriction, ATF4 induces various genes to adapt to cellular stresses [11]. However, the precise mechanisms by which ATF4 activates an appropriate gene in response to metabolic stresses are unclear.

In the present study, we identified the cellular signaling required for cell survival under various amino acid deprivation conditions. In particular, we found that the AKT activation required for cell survival was induced by GCN2/ATF4/REDD1 axis-mediated mTORC2 activation in response to amino acid deprivation in non-small cell lung cancer cells. Moreover, we found that knockout of REDD1 sensitizes cells cultured under glutamine deprivation conditions to radiotherapy. In conclusion, the GCN2/ATF4/REDD1 axis induced by amino acid deprivation promotes AKT activation which might be a potential target for cancer therapy.

## Results

### REDD1 and ATF4 expression under individual amino acid-deficient conditions

**Fig. 1:**
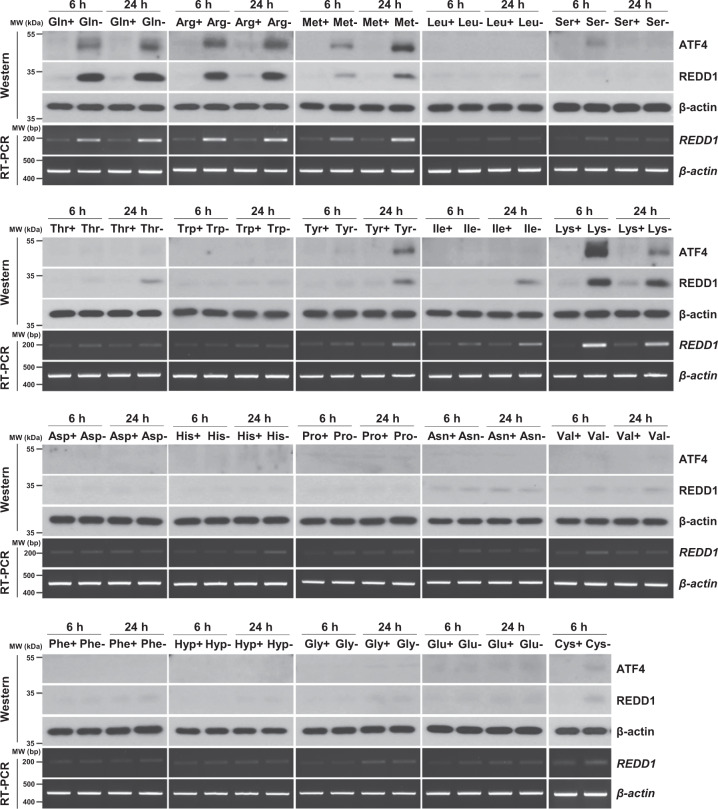
REDD1 and ATF4 expression in individual amino acid**-**deficient conditions. H1299 cells were deprived of each one of 20 amino acids in medium for 6 or 24 h. The levels of ATF4 and REDD1 protein and REDD1 mRNA were measured by western blot and RT-PCR analysis, respectively.

First, we investigated the effects of individual amino acid deprivation on REDD1 expression. H1299 non-small cell lung cancer (NSCLC) cells were cultured in medium deprived of each one of 20 amino acids, and REDD1 protein and mRNA expression levels were detected by western blot and RT-PCR analyses. Interestingly, the REDD1 protein and mRNA expression was greatly induced in cells deprived of glutamine, arginine, methionine, or lysine (Fig. 1). In cells deprived of threonine, tyrosine, isoleucine, or cysteine, the REDD1 protein and mRNA expression was also slightly induced. It was not possible to determine REDD1 protein and mRNA expression in cells deprived of cysteine for 24 h because of extensive cell death. Activating transcription factor 4 (ATF4) is the master regulator of amino acid metabolism and is activated by the lack of amino acids [9, 10]. Thus, we investigated ATF4 protein expression in the cells deprived of each amino acid. As shown in Fig. 1, ATF4 protein expression was increased in the cells lacking certain amino acids that induced REDD1 expression.

### The GCN2/ATF4 signaling pathway upregulates REDD1 expression upon glutamine or arginine deprivation conditions

**Fig. 2:**
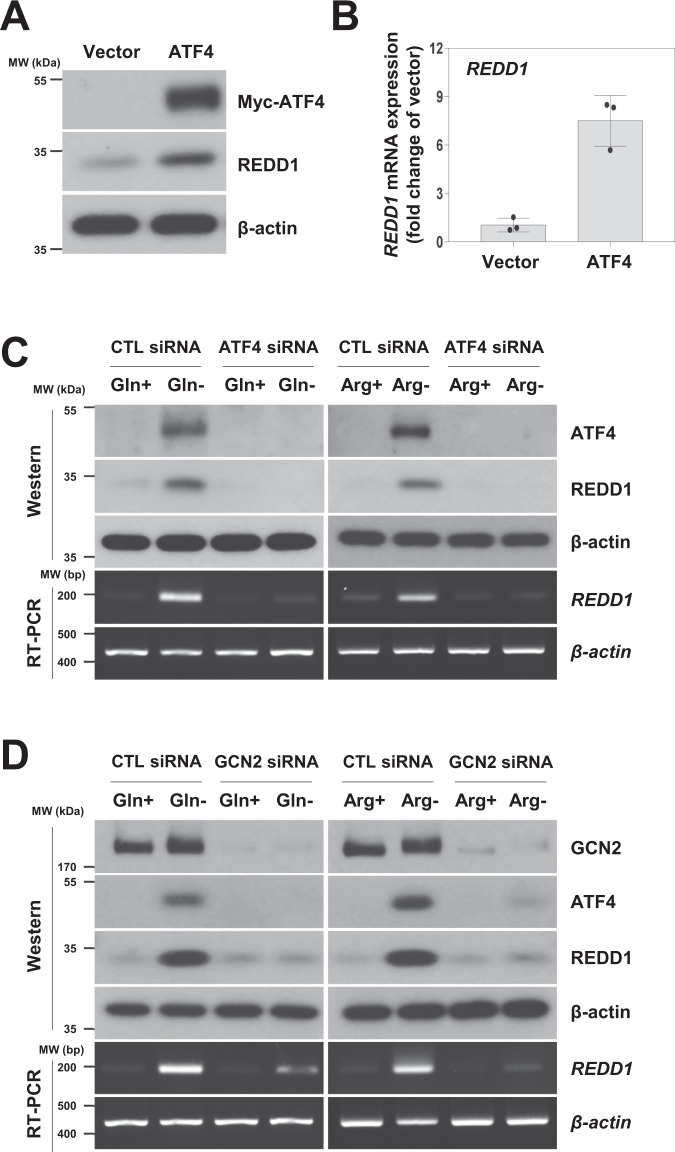
The GCN2/ATF4 signaling pathway upregulates REDD1 expression upon glutamine or arginine deprivation. **A**, **B** H1299 cells were transiently transfected with an empty vector (pEF) or a myc-tagged mATF4 (ATF4) plasmid for 24 h. The indicated protein and mRNA expression levels were measured by western blot and real-time PCR analyses (**A**, **B**). The real-time PCR results for each sample were analyzed according to the 2^−ΔΔCt^ method using β‑actin as the internal control. REDD1 mRNA expression is presented as the fold change relative to the control sample (*n* = 3). **C**, **D** H1299 cells were transfected with a control, ATF4 or GCN2 siRNA for 12 h and incubated in complete medium or medium lacking glutamine or arginine for 24 h. The indicated protein and mRNA expression levels were measured by western blot and RT-PCR analyses. CTL: control.

To investigate whether ATF4 is involved in the increased expression of REDD1, we examined REDD1 expression in H1299 cells transiently transfected with plasmids encoding Myc-ATF4. Compared with cells transfected with an empty vector, cells that overexpressed Myc-ATF4 exhibited increased levels of REDD1 mRNA and protein (Fig. 2A and B). These data suggest that REDD1 is a downstream target of ATF4, consistent with previous reports [12, 13]. Next, we further investigated whether ATF4 is critical for the upregulation of REDD1 expression in response to glutamine or arginine deprivation. Knocking down ATF4 by siRNA in H1299 cells abrogated ATF4 induction in cells in cultured in glutamine- or arginine-deprived medium (Fig. 2C). ATF4 depletion blocked REDD1 induction upon glutamine or arginine deprivation (Fig. 2C), suggesting that ATF4 activation is involved in the induction of REDD1 expression in response to glutamine and arginine deprivation.

In response to amino acid limitation, general control nonderepressible 2 (GCN2) kinase senses intracellular amino acid levels and activates ATF4 during metabolic adaptation [9]. Hence, we investigated whether GCN2 is involved in the expression of ATF4 and REDD1 in response to glutamine or arginine deprivation. Treatment with GCN2 siRNA suppressed the expression of ATF4 and REDD1 in the absence of glutamine or arginine in the medium (Fig. 2D). These data suggest that the GCN2/ATF4 pathway is important for the induction of REDD1 expression in response to glutamine or arginine deprivation.

### mTORC1 and mTORC2 activity under individual amino acid-deficient conditions

**Fig. 3:**
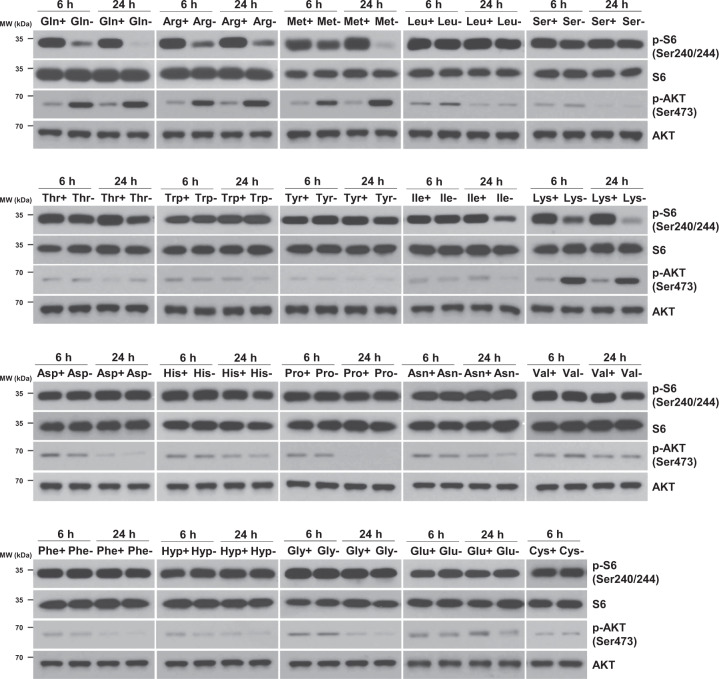
mTORC1 and mTORC2 activity under individual amino acid-deficient conditions. H1299 cells were deprived of each one of 20 amino acids in medium for 6 or 24 h. The levels of phosphorylated S6 (Ser240/244) and AKT (Ser473) were measured by western blot analysis.

It was reported that amino acid deficiency may differentially activate mechanistic target of rapamycin (mTOR) complexes 1 and 2 [14]. We investigated the effects of individual amino acid deprivation on the phosphorylation of the mTOR complex 1 (mTORC1) substrate S6 at Ser240/244 and the mTOR complex 2 (mTORC2) substrate AKT at Ser473. As shown in Fig. 3, deprivation of glutamine, arginine, methionine or lysine for 6 h or 24 h reduced S6 phosphorylation but induced AKT phosphorylation. Deprivation of isoleucine or histidine for 24 h reduced S6 phosphorylation but did not induce AKT phosphorylation. There was no dramatic change in S6 or AKT phosphorylation in the cells deprived of each one of the 14 other amino acids: leucine, serine, threonine, tryptophan, tyrosine, aspartic acid, proline, asparagine, valine, phenylalanine, hydroxyproline, glycine, glutamic acid, or cysteine. These data suggest that the withdrawal of glutamine, arginine, methionine or lysine in the H1299 NSCLC cells inhibits mTORC1 activity and induces mTORC2 activity.

### The GCN2/ATF4/REDD1 signaling axis contributes to mTORC1 inhibition and mTORC2 activation under glutamine or arginine deprivation conditions

**Fig. 4:**
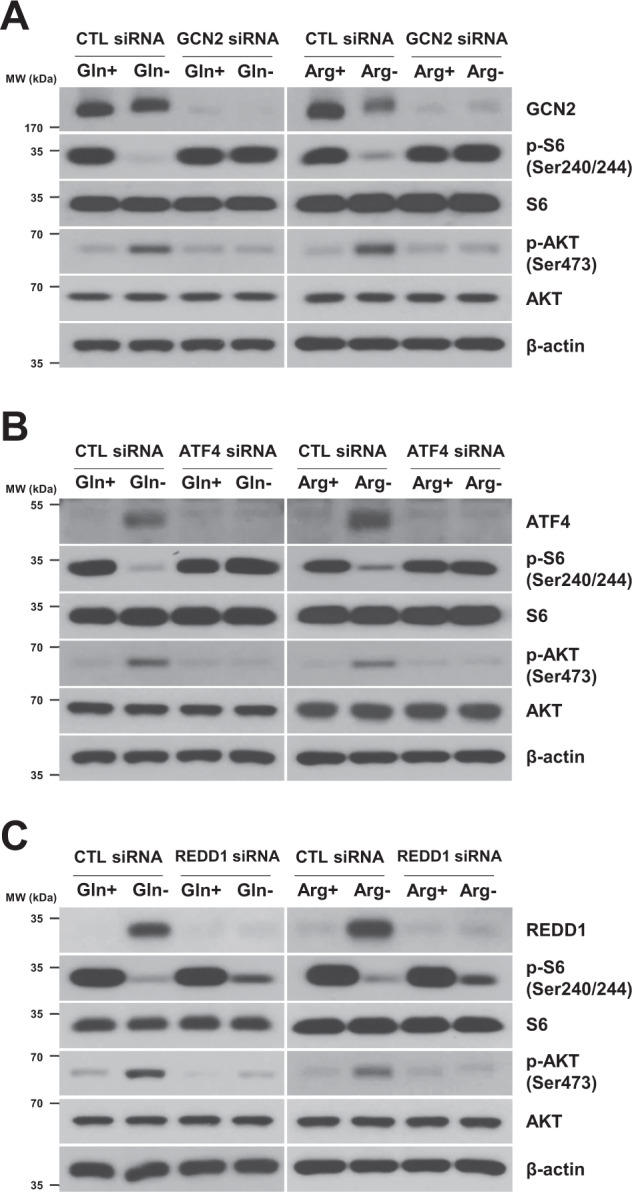
The GCN2/ATF4/REDD1 signaling axis contributes to mTORC1 inhibition and mTORC2 activation upon the deprivation of glutamine or arginine. **A**–**C** H1299 cells were transfected with a control, GCN2, ATF4, or REDD1 siRNA for 12 h and then subjected to glutamine or arginine deprivation for 24 h. The levels of the indicated proteins were measured by western blot analysis. CTL: control.

Next, we investigated whether the GCN2/ATF4/REDD1 axis is involved in mTORC1 inhibition and mTORC2 activation by glutamine or arginine deprivation. We knocked down GCN2, ATF4 or REDD1 expression by siRNA and then measured S6 and AKT phosphorylation in H1299 cells deprived of glutamine or arginine. Suppression of GCN2, ATF4 or REDD1 expression using the respective siRNA restored the decreased phosphorylation of S6 caused by glutamine or arginine deprivation (Fig. 4A–C). The GCN2, ATF4 or REDD1 siRNA attenuated glutamine- or arginine-induced AKT phosphorylation (Fig. 4A–C). These data suggested that the GCN2/ATF4/REDD1 signaling axis contributes to mTORC1 inhibition and mTORC2 activation in glutamine- or arginine-deprived cells.

### Replenishment of glutamine or arginine reverses the mTORC1 inhibition and mTORC2 activation induced by the deprivation of glutamine or arginine

**Fig. 5:**
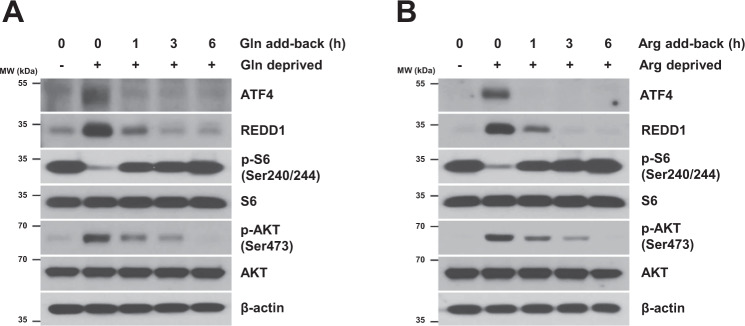
Replenishment of glutamine or arginine in the medium reverses the mTORC1 inhibition and mTORC2 activation induced by deprivation of glutamine or arginine. **A**, **B** H1299 cells deprived of glutamine or arginine for 24 h were supplemented with 300 mg/L glutamine or 200 mg/L arginine for the indicated times. The levels of the indicated proteins were measured by western blot analysis.

Next, we investigated ATF4/REDD1 and mTORC1/2 signaling by replenishing glutamine or arginine in glutamine- or arginine-deprived cell medium. As shown in Fig. 5, glutamine or arginine deprivation-mediated ATF4 and REDD1 induction was blocked when the medium was replenished with glutamine or arginine. The decrease in S6 phosphorylation and the increase in AKT phosphorylation in glutamine- or arginine-deprived cells were reversed after glutamine or arginine was added to medium, suggesting that replenishing glutamine or arginine reversed the decreased mTORC1 activity and increased mTORC2 activity in glutamine- or arginine-deprived NSCLC cells.

### Inhibition of mTORC2 activity suppresses AKT activation and enhances the inhibitory effects on the viability of glutamine-deprived cells

**Fig. 6:**
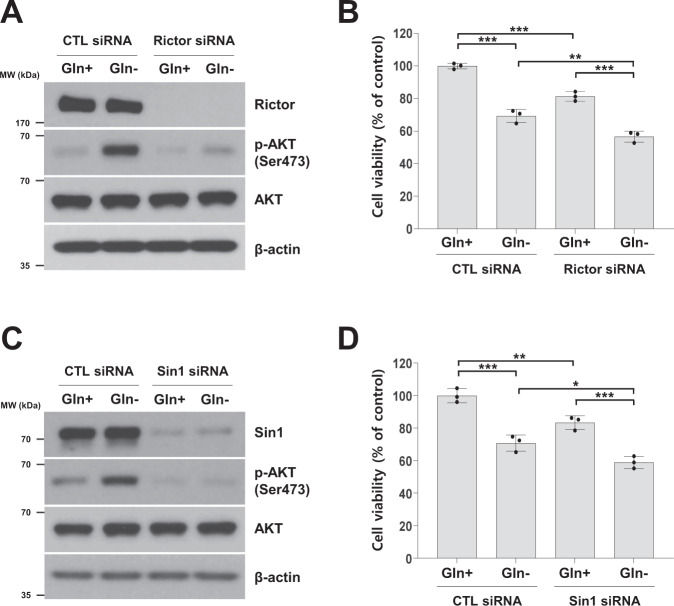
Inhibition of mTORC2 activity suppresses AKT activation and enhances the inhibitory effects on the viability of glutamine-deprived cells. **A**–**D** H1299 cells were transfected with a control, Rictor or Sin1 siRNA for 12 h and then subjected to glutamine deprivation for 24 h. **A**, **C** The level of phosphorylated AKT (Ser473) was measured by western blot analysis. **B**, **D** Cell viability was measured by MTT assay. The data are presented as the mean percentage of control ± SD relative to the control (*n* = 3; *p < 0.05; **p < 0.01; ***p < 0.001). CTL: control.

mTORC2 has been reported to regulate cell survival and proliferation through AKT phosphorylation at Ser473 [15, 16]. Rictor and Sin1, two components of mTORC2, are required for mTORC2-dependent phosphorylation of AKT [15–18]. Thus, we examined the AKT phosphorylation level and viability of H1299 cells deprived of glutamine following treatment with the siRNA against Rictor or Sin1. As expected, treatment with Rictor or Sin1 siRNA resulted in decreased levels of AKT phosphorylation in medium deprived of glutamine deprivation (Fig. 6A and C). Moreover, the Rictor and Sin1 siRNA significantly reduced the viability of the cells deprived of glutamine (Fig. 6B and D). These data suggested that amino acid deprivation-induced AKT phosphorylation occurs in an mTORC2-dependent manner.

### CRISPR-Cas9-mediated knockout of REDD1 suppresses AKT activation and enhances cell death in glutamine- or arginine-deprived cells

**Fig. 7:**
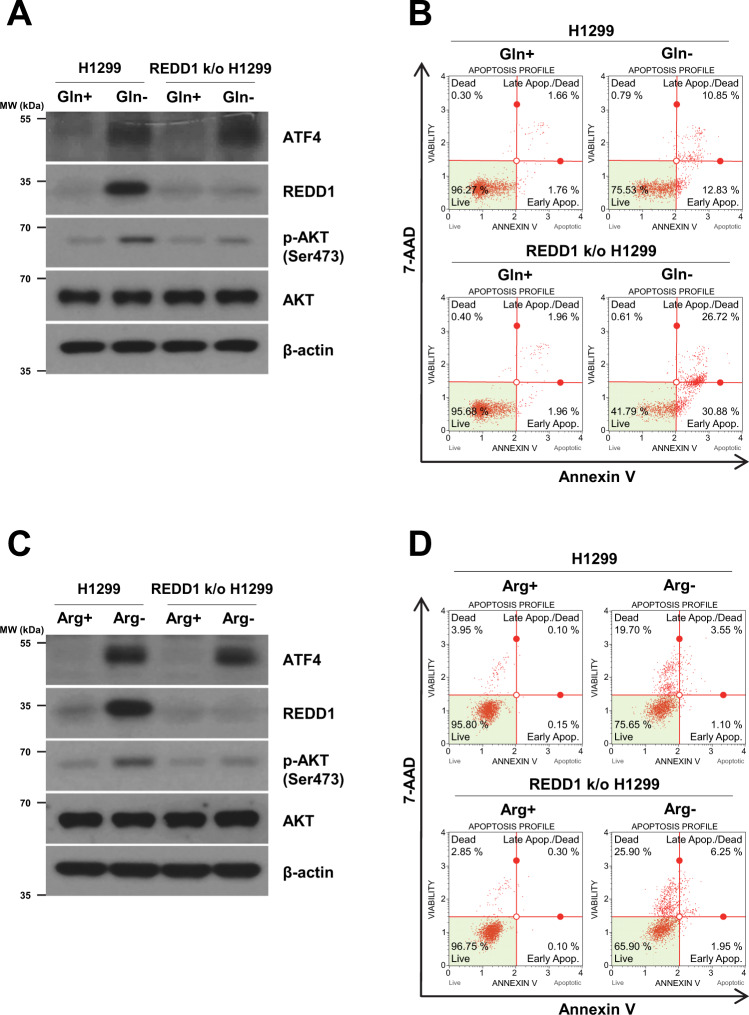
CRISPR-Cas9-mediated knockout of REDD1 suppresses AKT activation and enhances cell death in glutamine- or arginine-deprived cells. **A**–**D** H1299 and CRISPR/Cas9-mediated REDD1-knockout H1299 cells were incubated in complete medium or medium lacking glutamine or arginine for 24 h. **A**, **C** The levels of the indicated proteins were measured by western blot analysis. **B**, **D** Cell death was detected as the percentage of Annexin V and/or 7-AAD positive cells.

Next, we investigated whether disruption of REDD1 by CRISPR/Cas9 genome editing prevents AKT activation induced by glutamine or arginine deprivation. Although the high indel frequencies (>99.97%) of REDD1 gene were achieved (data not shown), the very weak expression of REDD1 protein was observed (Fig. 7A and C). The disruption to REDD1 expression inhibited the AKT phosphorylation induced by glutamine or arginine deprivation (Fig. 7A and C). ATF4 expression induced by glutamine or arginine deprivation was unchanged in CRISPR-Cas9-mediated REDD1-knockout cells (Fig. 7A and C). Next, we examined whether disruption of REDD1 leads to the induction of cell death in response to glutamine or arginine deprivation. As shown in Fig. 7B and D, the cell death rate was increased by glutamine or arginine deprivation in the culture medium of CRISPR/Cas9-mediated REDD1-knockout H1299 cells compared to that of H1299 cells. Disruption of REDD1 expression suppresses AKT activation and enhances cell death in glutamine- or arginine-deprived cells.

### Disruption of REDD1 or AKT activity enhances glutamine-deprived cell sensitivity to ionizing radiation

**Fig. 8:**
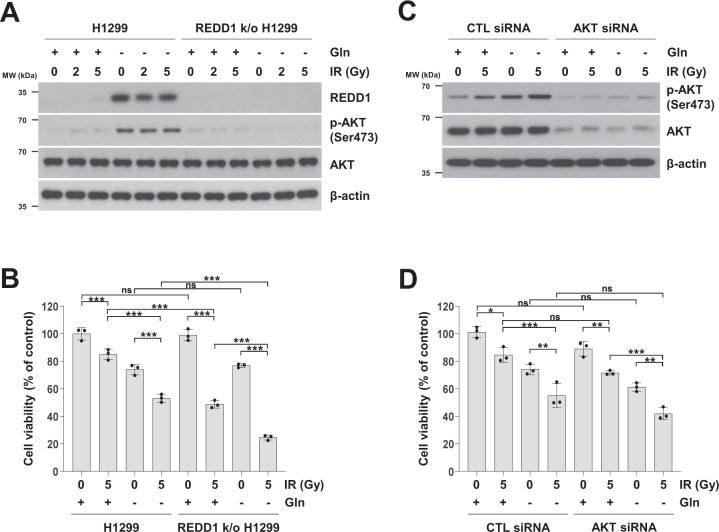
Disruption of REDD1 or AKT activity enhances cell sensitivity to ionizing radiation in glutamine-deprived cells. **A**, **B** H1299 and CRISPR/Cas9-mediated REDD1-knockout H1299 cells were exposed to the indicated dose of IR in the presence or absence of glutamine for 24 h. **C**, **D** H1299 cells were transiently transfected with a control or AKT siRNA for 12 h and subsequently exposed to 5 Gy of IR in the presence or absence of glutamine for 24 h. The levels of the indicated proteins were measured by western blot analysis (**A**, **C**). Cell viability was measured by MTT assay (**B**, **D**). The data are presented as the mean percentage of control ± SD relative to the control (*n* = 3; *p < 0.05; **p < 0.01; ***p < 0.001; ns, not significantly different). CTL: control, IR: ionizing radiation.

As REDD1 plays a role in AKT activation in response to glutamine deprivation, we investigated whether disruption of REDD1-enhanced cell sensitivity to ionizing radiation (IR) under glutamine deprivation. IR or co-treatment with IR and glutamine deprivation induced AKT phosphorylation (Fig. 8A and C). The viability of cells treated with a 5 Gy dose of IR was reduced by 20%, and co-treatment with IR and glutamine deprivation decreased cell viability to ~ 55% (Fig. 8B and D). Disruption of REDD1 activity inhibited AKT phosphorylation by co-treatment with IR and glutamine deprivation and further enhanced the IR sensitivity of glutamine-deprived cells (Fig. 8A and B). Knockdown of AKT also enhanced cell sensitivity to IR under glutamine deprivation conditions (Fig. 8D). Our results suggested that suppression of REDD1 and AKT in response to glutamine deprivation enhances cell sensitivity to IR, and the targeting of REDD1 and AKT under glutamine deprivation conditions may be an effective approach to enhancing lung cancer cell sensitivity to IR treatment.

## Discussion

Cancer cells are characterized by high proliferation rates, which requires a constant and ample supply of nutrients to support the anabolism that drives the biosynthesis of macromolecules, such as proteins, fatty acids, cholesterol, nucleotides, and other cellular building blocks [19, 20]. The overt dependence of cancer cells on an unperturbed nutrient supply has prompted widespread research into amino acid restriction as a potential cancer treatment strategy [19, 20]. Limiting amino acids may enable selective targeting of highly proliferative cancer cells [19]. However, owing to rapid signaling and metabolic reprogramming in cancer cells, the prospects for the success of amino acid restriction approaches are unclear. This study revealed the axis of cancer cell adaptation to amino acid deprivation and investigated mechanisms that may be targeted for enhancing the therapeutic efficacy of amino acid deprivation strategies.

Mechanistic target of rapamycin complex 1 (mTORC1) is a master regulator that promotes anabolism [21]. Amino acids promote mTORC1 lysosomal localization and subsequent activation [22, 23]. The molecular mechanisms by which individual amino acids activate mTORC1 are beginning to be revealed. In recent studies, amino acid sensors that mediate amino acid-induced mTORC1 activation have been identified, such as Sestrin for leucine, Castor for arginine, and SAMTOR for S-adenosylmethionine [24–26]. In this study, we evaluated the effects of a single amino acid on mTORC1 activity in H1299 non-small cell lung cancer (NSCLC) cells. Amino acid withdrawal experiments were performed in which an individual amino acid was removed from the medium, and the phosphorylation of the mTORC1 substrate S6 was analyzed. Utilizing the 20 standard amino acids, we found that deprivation of glutamine, arginine, methionine or lysine significantly inhibited mTORC1 activity. Deprivation of these amino acids induced the expression of REDD1, a negative regulator of mTORC1. A recent study reported that upregulated REDD1 expression is involved in resuppression of mTORC1 during prolonged leucine deprivation [27]. However, in our experimental system, leucine deprivation did not affect REDD1 expression or mTORC1 activity. These findings suggest that the ability of a given amino acid to regulate mTORC1 activity might differ according to cell type and severity of the stress encountered.

Activating transcription factor 4 (ATF4) is a basic leucine zipper transcription factor that is selectively translated in response to specific forms of cellular stress to induce the expression of genes involved in adaptation to stress [28]. The adaptive program is initiated by general control nonderepressible 2 (GCN2) upon amino acid deprivation. Our study and other studies have reported that ATF4 is critical for the increased expression of REDD1 [12, 13]. In our current study, ATF4 protein expression was increased in cells cultured in medium lacking glutamine, arginine, methionine or lysine, which induced REDD1 expression. ATF4-silenced cells displayed decreased REDD1 expression and sustained mTORC1 activity upon deprivation of glutamine or arginine, suggesting that ATF4 is required for REDD1 induction and mTORC1 inhibition in response to amino acid deprivation. Knockdown of GCN2 abrogated ATF4 and REDD1 induction and led to sustained mTORC1 activity upon deprivation of glutamine or arginine, indicating that GCN2/ATF4 signaling is required for REDD1 induction and mTORC1 inhibition in response to amino acid deprivation. Inhibition of REDD1 expression using siRNA prevented mTORC1 inhibition caused by glutamine or arginine deprivation, indicating that REDD1 induction is required for mTORC1 inhibition under amino acid deprivation. These data suggest that REDD1 is a critical effector of GCN2/ATF4 signaling and is required to sustain mTORC1 inhibition triggered by amino acid deprivation.

Interestingly, deprivation of glutamine, arginine, methionine, or lysine induced AKT phosphorylation at Ser473. mTORC2 has been shown to fully activate AKT by phosphorylating Ser473 to promote cell survival and proliferation [16]. siRNA-mediated depletion of Rictor or Sin1, a regulatory protein of the mTORC2 complex, abrogated AKT phosphorylation and enhanced cell sensitivity to glutamine or arginine deprivation, suggesting that mTORC2-mediated AKT activation governs cancer cell survival under amino acid deprivation. GCN2-, ATF4-, or REDD1-silenced cells exhibited decreased AKT phosphorylation at Ser473 under glutamine or arginine deprivation conditions, suggesting that mTORC2 activation in the context of amino acid deprivation depends on GCN2, ATF4, and REDD1 expression. AKT phosphorylation at Ser473 in response to glutamine or arginine deprivation was also decreased in CRISPR/Cas9-mediated REDD1-knockout NSCLC cells. Disruption of REDD1 activity enhanced cell death in response to glutamine or arginine deprivation. Furthermore, knockout of REDD1 sensitized cells cultured in glutamine-deprived medium to radiotherapy. These data suggested that mTORC2-mediated AKT activation by REDD1 under amino acid-insufficient conditions is required for maintaining cell survival.

**Fig. 9:**
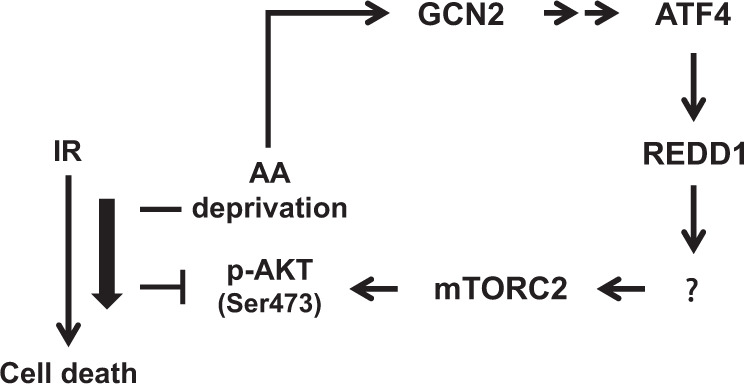
Proposed mechanism by which amino acid deprivation induces AKT activation necessary for non-small cell lung cancer cell survival. Amino acid deprivation induces REDD1 expression by GCN2/ATF4 pathway activation. REDD1 induction by amino acid deprivation in turn activates the mTORC2/AKT axis, which is required for cell survival. Disruption of REDD1 inhibits AKT phosphorylation by IR and glutamine deprivation and enhances glutamine-deprived cell sensitivity to IR. AA: amino acid, IR: ionizing radiation.

In summary, we found that REDD1 induction by GCN2/ATF4 pathway activation upon amino acid deprivation activates the mTORC2/AKT axis, which is required for cell survival (Fig. 9). Also, we observed that disruption of REDD1 inhibits AKT phosphorylation by IR and glutamine deprivation and further enhanced the IR sensitivity of glutamine-deprived cells (Fig. 9). REDD1 is integrated into the mTORC2/AKT signaling axis and protects cells against energy-related stress. A future study will be required to determine how REDD1 activates mTORC2 under amino acid deprivation conditions.

## Materials and methods

### Cell culture and reagents

H1299 human non-small cell lung cancer cells were obtained from the American Type Culture Collection (Manassas, VA, USA), and were maintained in RPMI 1640 medium (#LM011-01, Welgene, Gyeongsangbuk-do, Republic of Korea) containing 10% fetal bovine serum (#16000-044, Gibco; Thermo Fisher Scientific, Waltham, MA, USA) at 37 °C and 5% CO_2_. The RPMI medium was deprived of one amino acid and each of these amino acid-deprived RPMI 1640 media was supplemented with 10% dialyzed fetal bovine serum (10,000 MW cut-off, #26400-044, Gibco; Thermo Fisher Scientific). Thiazolyl blue tetrazolium bromide (MTT) was purchased from Sigma-Aldrich (#M5655, Merck KGaA, Darmstadt, Germany). ^137^Cesium was used as a source of γ-radiation (Atomic Energy of Canada Limited, Chalk River, ON, Canada).

### Measurement of cell viability

Cell viability was assessed by measuring the mitochondrial conversion of MTT. The proportion of converted MTT was calculated by measuring cell absorbance at 570 nm. The results are expressed as the percent reduction in MTT under the assumption that the absorbance of the control cells was 100%. Each MTT experiment was repeated 3 times.

### Detection of cell death

Cell death was assayed using a Muse Annexin V and dead cell kit (#MCH100105, Millipore; Merck KGaA) according to the manufacturer’s instructions. Cells were suspended in RPMI 1640 medium with 1% bovine serum albumin and mixed with Muse Annexin V and dead cell reagent. The cells were then incubated in the dark for 20 min at room temperature, and the cell death rate was measured using a Muse Cell analyser (Millipore; Merck KGaA). Cells were characterized into four groups: Live (Annexin V negative, 7-AAD negative), early apoptotic (Annexin V positive, 7-AAD negative), late apoptotic (Annexin V positive, 7-AAD positive), and necrotic (Annexin V negative, 7-AAD positive). Cell death was detected as the percentage of Annexin V and/or 7-AAD positive cells.

### RNA extraction and reverse transcription PCR

RNA was isolated from cells using TRIzol reagent according to the manufacturer’s instructions (#15596-026, Invitrogen; Thermo Fisher Scientific). cDNA primed with oligo dT was prepared from 2 μg of total RNA using M-MLV reverse transcriptase (#28025-021, Invitrogen; Thermo Fisher Scientific). The following specific primers were used for PCR: *REDD1* (5’- AGCCAGTTGGTAAGCCAGG-3’ and 5’- GCCAGAGTCGTGAGTCCAG-3’, a 199 bp product) [29] and *β-actin* (5’- GGATTCCTATGTGGGCGACGA-3’ and 5’- CGCTCGGTGAGGATCTTCATG-3’; a 438 bp product) [29]. The PCR products were visualized on a 2% agarose-TAE gel containing ethidium bromide.

### Real-time PCR

Real-time PCR was performed using TaqMan gene expression assay probes (Applied Biosystems; Thermo Fisher Scientific) for *REDD1* (assay ID: Hs01111681_g1) mRNA quantification. *β-actin* (assay ID: Hs01060665_g1) was used as the internal control. Quantitative real-time PCR was performed using an ABI 7500 Real Time PCR system (Applied Biosystems; Thermo Fisher Scientific). The fold change in gene expression was determined by the comparative CT (2^–ΔΔCT^) method.

### Transient transfection

The expression plasmid encoding mouse wild-type ATF4 (pEF/mATF4-myc) was kindly provided by Dr. Jawed Alam [30]. GCN2 (#sc-45644), ATF4 (#sc-35112), REDD1 (#sc-45806), Rictor (#sc-61478), Sin1 (#sc-60984) and control (#sc-37007) siRNAs were purchased from Santa Cruz Biotechnology (Dallas, TX, USA). Transfection with plasmids and siRNAs was performed using Lipofectamine Plus (#15338) or Lipofectamine RNAiMAX (#13778), respectively, according to each manufacturer’s instructions (Invitrogen: Thermo Fisher Scientific).

### REDD1-knockout H1299 cell line

The REDD1-knockout H1299 cell line was generated by nSAGE Inc. (Incheon, Republic of Korea). Briefly, H1299 cells were transfected with five different sgRNAs (SG1: 5’-TCCTCACCATGCCTAGCCTTTGG-3’, SG2: 5’-CCTCACCATGCCTAGCCTTTGGG-3’, SG3: 5’-GAGAAGCGGTCCCAAAGGCTAGG-3’, SG4: 5’-ACGACGAGAAGCGGTCCCAAAGG-3’, SG5: 5’-AGGTGGACGACGACGAGAAGCGG-3’) and pRGEN-Cas9-CMV/T7-Puro-RFP using Lipofectamine 2000 (#11668, Invitrogen; Thermo Fisher Scientific). After 72 h of incubation, the cells were harvested for gDNA isolation for NGS (iSeq 100, Illumina, San Diego, CA, USA) analysis and further expansion. The efficiencies of the designated sgRNAs were evaluated with their indel frequency in the total cell population. The cell line demonstrating the highest indel frequency (>99.0%) and pattern of out-of-frame mutations was isolated by single clonal selection. The knockout efficiency was verified by western blotting with an anti-REDD1 antibody (#10638-1-AP, Proteintech Group, Rosemont, IL, USA).

### Western blot analysis

Equal amounts of total cellular protein were separated by 6~12% sodium dodecyl sulfate-polyacrylamide gels and transferred to nitrocellulose membranes followed by immunoblotting with the specified primary and horseradish peroxidase-conjugated secondary antibodies. The following antibodies were used: anti-ATF4 antibody (#sc-200) obtained from Santa Cruz Biotechnology; anti-REDD1 (#10638-1-AP) obtained from Proteintech Group; anti-GCN2 (#3302), anti-AKT (#9272), anti-p-AKT (Ser473; #9271), anti-Rictor (#2114), anti-Sin1 (#12860), anti-S6 (#2217), and anti-p-S6 (Ser240/244; #5364) antibodies obtained from Cell Signaling Technology (Beverly, MA, USA); and anti-β-actin (#A5316) antibody obtained from Sigma-Aldrich (Merck KGaA).

### Statistical analysis

The data are expressed as the mean ± standard deviation (SD) of three independent experiments. Statistical differences were measured by one-way ANOVA followed by Tukey’s test using GraphPad Prism software (version 9.0, San Diego, CA, USA). Differences with *p* < 0.05 were considered statistically significant.

## Supplementary information

Reproducibility checklist
